# The Impact of Changes in Corneal Back Surface Astigmatism on the Residual Astigmatic Refractive Error following Routine Uncomplicated Phacoemulsification

**DOI:** 10.1155/2020/7395081

**Published:** 2020-07-22

**Authors:** Larysa Tutchenko, Sudi Patel, Oleksiy Voytsekhivskyy, Mykhailo Skovron, Olha Horak

**Affiliations:** ^1^Department of Ophthalmology, Shupyk National Medical Academy of Postgraduate Education, Kyiv, Ukraine; ^2^Kyiv City Clinical Ophthalmological Hospital “Eye Microsurgical Center”, Kyiv, Ukraine; ^3^NHS National Services Scotland, Edinburgh, UK

## Abstract

**Purpose:**

To determine the significance of any association between intersessional changes in ocular residual astigmatism (RA) and astigmatism at corneal front (FSA) and back (BSA) surfaces following uneventful routine phacoemulsification.

**Methods:**

Astigmatism was evaluated by autorefractometry and subjective refraction and at both the corneal surfaces with Orbscan II™ (Bausch & Lomb) over central 3 mm and 5 mm optical zones at 1, 2, and 3 months after routine phacoemulsification in 103 patients implanted with monofocal nontoric intraocular lenses (IOLs, one eye/patient). Data were subjected to vector analysis to determine the actual change (Δ) in astigmatism (power and axis) for the refractive and Orbscan II findings.

**Results:**

The number of cases that attended where ΔRA was ≥0.50 DC between 1 and 2 months was 52 by autorefractometry and 36 by subjective refraction and between 2 and 3 months was 24 by autorefractometry and 19 by subjective refraction. Vector analysis revealed significant correlations between ΔFSA and ΔRA for data obtained by autorefractometry but not by subjective refraction. At all times, ΔBSA was greater than ΔFSA (*p* < 0.01). Key findings for ΔBSA values over the central 3 mm zone were between (A) the sine of the axis of ΔRA (*y*) and sine of the axis of ΔBSA (*x*) for the data obtained by autorefractometry (between 1 and 2 months, *y* = 0.749 − 0.303*x*, *r* = 0.299, *n* = 52, *p*=0.031) and subjective refraction (between 2 and 3 months, *y* = 0.6614 − 0.4755*x*, *r* = 0.474, *n* = 19, *p*=0.040) and (B) ΔRA (*y*) and ΔBSA (*x*) powers between 2 and 3 months postoperatively for the data obtained by autorefractometry (ΔRA = 0.118 ΔBSA + 0.681 *r* = 0.467, *n* = 24, *p*=0.021) and subjective refraction (ΔRA = 0.072 ΔBSA + 0.545 *r* = 0.510, *n* = 19, *p*=0.026).

**Conclusion:**

Changes in the ocular residual refractive astigmatic error after implanting a monofocal nontoric IOL are associated with changes in astigmatism at the back surface of the cornea within the central optical zone.

## 1. Introduction

The current procedures for calculating intraocular lens (IOL) power have reduced the magnitude of postoperative refractive errors in pseudophakic eyes. However, unexpected astigmatic surprises can still be encountered after phacoemulsification. Changes in IOL position, tilt, corneal curvature, and the cumulative errors associated with IOL power estimation are likely sources of unexpected refractive surprises. An unpredicted change in astigmatism at the front surface of the cornea will affect ocular residual astigmatism following phacoemulsification. Could unexpected modulation of corneal back surface astigmatism contribute to changes in ocular residual astigmatism between one time point and the next? An understanding of the corneal back surface astigmatism is important in relation to toric IOL power selection [1–3]. Ignoring corneal back surface astigmatism can lead to errors in estimating the surgically induced astigmatism and IOL power selection [1–10]. Two recent studies found that ocular residual astigmatism >1.00 DC occurred in approximately 20% of eyes at 3 months postoperatively [11, 12]. These two investigations were based on quite different patient groups, toric IOL designs, surgical procedures, and IOL power algorithms. Nevertheless, both groups reported similar outcomes and both precluded exact details of corneal back surface astigmatism when selecting toric IOL powers.

If corneal back surface astigmatism plays an important role in toric IOL power selection, then could changes in astigmatism at this surface have impact on changes in ocular residual astigmatism in cases implanted with nontoric IOLs? A brief review of the literature shows that the mean radius of curvature at this surface ranges from 5.8 mm to 6.8 mm [1, 13–16]. Simple ray tracing through classic model eyes (e.g., Gullstrand no. 2) predicts the change in the radius of this surface from 6 mm to 7 mm that alters the refractive error by 1.00 D [13]. The test-retest reliability of subjective refraction ranges from ±0.34 D to ±0.51 D, and refractive astigmatism <0.50 DC has a negligible effect on visual acuity [17–21]. Therefore, changes in refractive astigmatism are significant when ≥0.50 DC. Corneal hydration can alter following phacoemulsification, and coupled with changes in the distribution of corneal mass, it could impact on the radii of both corneal surfaces [22–25]. The precise mechanism of any change in corneal mass distribution could lead to unexpected astigmatic refractive surprises.

Is there an empirical association between changes in astigmatism at both corneal surfaces when a change in ocular refractive astigmatism occurs between one time and another after implantation of a monofocal nontoric IOL?

The aim of this study was to provide answers to the question posed by; first, monitoring astigmatism by objective and subjective refraction and the astigmatism at both corneal surfaces, and second, subjecting all astigmatic data to vector analysis to calculate the actual change in astigmatism in each component in cases that underwent routine uncomplicated phacoemulsification.

## 2. Materials and Methods

### 2.1. Measurement of Corneal Surfaces and Refractive Astigmatism

Orbscan II (Bausch & Lomb, Rochester, NY) provides axial dioptric power maps of both corneal surfaces over the central optical zone diameters of 3 mm and 5 mm and the average pupil diameter under the prevailing conditions. This device is a placido disc scanning slit-lamp system designed to retrieve data from 9000 locations over the cornea [26]. The scanning slit provides the numerical separation between the two corneal surfaces at each location. These data, in conjunction with the dioptric power maps generated for the front surface, are used to construct an axial dioptric power map of the back surface. The algorithm used is an extension of earlier methods [13, 25]. Calculation of corneal back surface powers is based on refractive index values of 1.376 for the cornea and 1.336 for the aqueous humour. The data from the multiple power maps are averaged to provide astigmatic power, and axis, values over two corneal chord diameters (3 mm and 5 mm). Full descriptions and functionality of Orbscan II are provided elsewhere [26, 27]. This is a long-standing instrument proven to be useful for examining the corneal surfaces in a wide range of studies [11, 28–33]. Refractive astigmatism was determined by autorefractometry and by routine subjective refraction.

### 2.2. Calculating Change in Astigmatism

Suppose astigmatism changed from +1.00 DC × 155 to +1.50 DC × 155 between times T_1_ and T_2_, then the change in astigmatism was +0.50 DC × 155. Simple subtraction cannot be used when there is a change in the axis of astigmatism. Methods based on vector analysis must be employed for the numerical assessment when changes occur in both the power and axis. The astigmatic data obtained by refraction and Orbscan II were subjected to analysis using two methods. First, a simple graphical adjustment [34] that produces results identical to those produced using the method advanced by Alpins [35], and second, the technique proposed by Thibos et al. [36]. The first procedure renders astigmatic data in a polar format, while the second expresses the same data in a Cartesian format. The first method calculates the exact difference, in both power and axis, of any change in astigmatism between times T_1_ and T_2_. The second method requires computation of J_0_ and J_45_ vectors as described elsewhere [34]. This process brings together individual pairs of astigmatic power and axis values into single figures (J_0_ and J_45_). Sets of J_0_ and J_45_ values are amenable to relatively simple statistical analysis. Two algorithms were used to calculate the two sets of vectors even though it is possible to derive the results of the first procedure from the second. The changes in astigmatism over the corneal surfaces, and by both objective and subjective refraction, were calculated for the periods between 1 and 2 months and between 2 and 3 months postoperatively.

### 2.3. Study Design

This was a prospective, consecutive, partially masked, single-centre, observational study adhering to the tenets of the Declaration of Helsinki and approved by the local ethics committee. All patients gave signed consent after the purpose and procedures of the study were fully explained. Measurements were obtained from each patient on a consecutive, case-by-case, basis.

### 2.4. Exclusion Criteria

None of the patients enrolled had any history of previous ocular surgery, contact lens wear, high refractive error, corneas thinner than 545 *μ*m, unusual corneal topography (at the front or back surface of the cornea), corneal opacities, and active or previous conditions linked to either the anterior or posterior segment. All had a need for routine cataract surgery only.

### 2.5. IOL Power Selection

Biometry was performed by one examiner using an IOL Master 700 SWEPT source OCT-biometer, software version 1.70 (Carl Zeiss, Meditec AG, Jena). Eight IOL power formulae of the 3rd and 4th generation were used: Barrett Universal II, Haigis, Hoffer Q, Holladay 1, Holladay 2, SRK/T, T2, and VRF. All except T2 and VRF were part of the IOL Master 700 software version 1.70. The T2 formula is a modified and enhanced version of the most popular SRK/T formula resulting in significant improvements of prediction accuracy and is available from the author's website as an Excel spreadsheet (Microsoft, Redmond, WA), T2 formula Calculator V.1.3. In comparison to SRK/T, the T2 formula leads to a 10% improvement in the prediction accuracy of refractive outcomes [37]. As an additional feature, a recently published VRF formula was used for all our IOL power calculations. This formula has been validated for an extensive range of axial lengths, and it is available as part of the ViOL Commander Software version 2.0.0.0 [38]. The most appropriate and suitable formula was chosen by the examiner depending on the parameters of the eye and personal experience. For short eyes, the Haigis, Hoffer Q, and VRF formulae were used; for eyes with medium axial lengths, Holladay 1 and VRF were used; for eyes with medium to long axial lengths, Holladay 1, Holladay 2, and T2 were used; and for long eyes, Barrett Universal II and SRK/T were used. The IOL selected for implantation in each case was a hydrophobic acrylic 1-piece monofocal nontoric lens of either an aspheric (SN60WF, Alcon Surgical, Inc.) or spherical (SN60AT, Alcon Surgical, Inc.) design.

### 2.6. Description of Preoperative Preparation, Surgery, and Postoperative Treatment

The horizontal axis on the cornea, of the eye scheduled for treatment, was marked by one examiner (LT) using a slit-lamp marking technique under topical anaesthesia prior to pupil dilation. The slit-lamp beam width was adjusted to its minimum visible setting, turned, and horizontally aligned over the pupil centre. The slit-lamp was moved to the contralateral eye to ensure both eyes were positioned along a common axis. When the first Purkinje images in both eyes were aligned at the same height, the slit-lamp was then moved over to the eye scheduled for treatment without changing the height of the beam. The horizontal axis was scratch marked on the cornea at the limbal 3 and 9 o'clock positions with a 30-gauge sterile needle and staining of corneal microabrasions with the 2% collargoli solution (colloidal silver solution). Surgery was performed by one surgeon (LT) under topical anaesthesia through a 2.2 mm self-sealing clear corneal incision. In all cases, a corneal tunnel was made at 12 o'clock using a Mendez ring with reference to the preoperative markings. A 1.2 mm paracenthesis was made at 3 and 9 o'clock with reference to the preoperative marks. A 5.0 mm circular capsulorhexis was followed by lens hydrodissection, phacoemulsification, and bimanual cortex removal using the Infinity Vision System (Alcon Surgical, Inc.). A hydrophobic acrylic 1-piece monofocal IOL was placed in the capsular bag, and the surgical wound was closed by stromal hydration. Surgery was completed with injections of dexamethasone (subconjunctival) and betamethasone (parabulbar). Postoperative treatment involved drops of levofloxacin, dexamethasone, and indomethacin with gradual tapering off, dexpanthenol gel, and a combination of trehalose and hyaluronic acid. IOP was within normal limits at all examinations postoperatively.

### 2.7. Pre- and Postoperative Assessment of Corneal Topography, Refractive Astigmatism, and Pupil Diameter

A recently serviced and calibrated Orbscan II (Bausch & Lomb, Rochester, NY, version 3.2) set at an acoustic equivalent correction of 0.92, for estimating pachymetry values, was used on all cases. Dioptric power maps, mean astigmatic powers and axes, over the central 3 mm and 5 mm optical zones of both corneal surfaces, and the pupil diameter were recorded in accordance with the user's handbook. The data for corneal front and back surface astigmatic powers (DC) and axes (°) over the 3 and 5 mm zones were recorded for analysis. The refractive astigmatism was determined objectively using a single calibrated autorefractometer (Tomey RT-7000, Tomey Corp, Tokyo, Japan) followed by routine subjective refraction. All cases were checked preoperatively and at one, two, and three months postoperatively.

### 2.8. Data and Statistical Analysis

All data were stored on an Excel spread sheet (Microsoft, Redmond, WA), and all refractive and corneal astigmatic data were subjected to vector analysis. Those cases where, according to vector analysis, the change in the ocular residual astigmatism was less than 0.50 DC were excluded from further analysis. Preliminary exploratory analysis using the trigonometric functions of the astigmatic axes demonstrated that correlations were the most powerful for the sine function. This was not unreasonable considering that the sine remains positive over the range 0°–180°. Hence, to avoid the masking of significant associations, it was decided to include only the sine of axis angles in linear regression analyses. The results were analysed to determine the significance of any of the following:

- Difference between the two IOL designs in relation to the magnitude of changes in astigmatism over the postoperative periods (unpaired *t*-test)
- Difference between changes in corneal front and back surface astigmatic powers over the central 3 mm and 5 mm zones (paired *t*-test)
- Apparent association between the change in corneal front and back surface astigmatic powers and the change in unexpected ocular residual astigmatic power determined by objective and subjective refraction (Pearson correlation coefficient, *r*)
- Apparent association between the change in the axis of corneal front and back surface astigmatism and change in the axis of unexpected ocular residual astigmatism determined by objective and subjective refraction (Pearson correlation coefficient, *r*)
- Correlation between the astigmatic vector (J_0_ and J_45_) describing the change in astigmatism by refraction and the corresponding astigmatic vector value describing the change in corneal front and back surface astigmatism (Pearson correlation coefficient, *r*)

The significance level was set at *p* < 0.05.

## 3. Results

**Figure 1:**
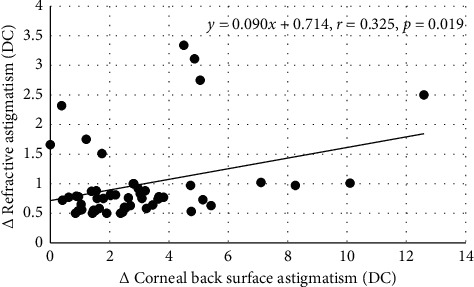
Change in astigmatism at the corneal back surface over the central 3 mm zone (*x* axis) and the corresponding change in residual refractive astigmatic error determined by autorefractometry (*y* axis) between 1 and 2 months postoperatively. All units are in diopters.

**Figure 2:**
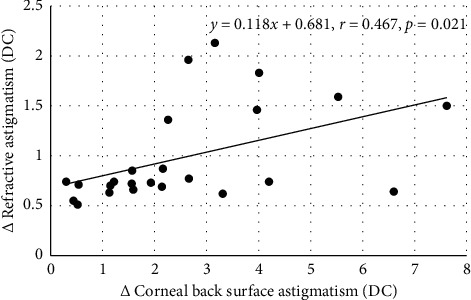
Change in astigmatism at the corneal back surface over the central 3 mm zone (*x* axis) and the corresponding change in residual refractive astigmatic error determined by autorefractometry (*y* axis) between 2 and 3 months postoperatively. All units are in diopters.

**Figure 3:**
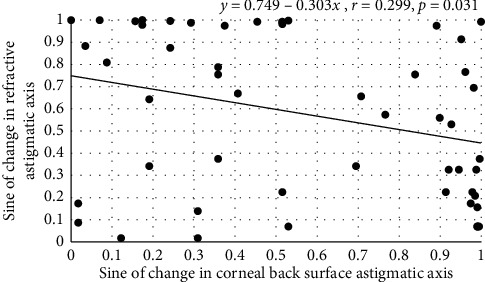
Change in the sine of the axis of astigmatism at the corneal back surface over the central 3 mm zone (*x* axis) and the corresponding change in the sine of the axis of residual refractive astigmatic error determined by autorefractometry (*y* axis) between 1 and 2 months postoperatively. All units are sine of axis in degrees (°).

One hundred three eyes (of 103 patients) were implanted with monofocal nontoric IOLs. The patients consisted of 50 females and 53 males of mean age 69.5 ± 10.4 years (range 40–90 years). The mean (±SD) pupil size was 3.39 mm ± 0.68 mm (range 1.6–4.7 mm). There were no complications associated with surgery. Ninety-two returned for follow up at one month, eighty-three at two months, and forty-nine at 3 months. Fifty-two patients were implanted with the aspheric SN60WF (group I), and 51 were implanted with the spherical SN60AT (group II). Summaries of the chief results are shown in Tables 1 and 2 and Figures 1–3. All astigmatic values are reported in a positive format.

### 3.1. Comparison of IOL Designs

The actual change in ocular residual astigmatism (RA) determined by objective refraction was ≥0.50 DC in 23 group I and 29 group II cases between 1 and 2 months postoperatively. Vector analysis revealed that the mean (±SD) changes of astigmatic powers were 0.93 DC (±0.62, 95% CI 0.66–1.20) in group I and 0.97 DC (±0.79, 95% CI 0.69–1.27) in group II. The difference between the groups was not significant (*p*=0.760). Between 2 and 3 months postoperatively, the respective figures were 1.04 DC (±0.16, 95% CI 0.93–1.16, *n* = 9) in group I and 1.08 DC (±0.52, 95% CI 0.79–1.37, *n* = 15) in group II. The difference was not significant (*p*=0.755). The actual change in RA determined by subjective refraction data was ≥0.50 DC in 15 group I and 21 group II cases between 1 and 2 months postoperatively. Vector analysis revealed that the mean (±SD) changes of astigmatic powers were 1.03 DC (±0.60, 95% CI 0.69–1.36) in group I and 1.04 DC (±0.70, 95% CI 0.72–1.35) in group II. The difference was not significant (*p*=0.799). Between 2 and 3 months postoperatively, the respective figures were 0.66 DC (±0.29, 95% CI 0.39–0.93, *n* = 7) in group I and 0.89 DC (±0.55, 95% CI 0.54–1.24, *n* = 12) in group II. The difference was not significant (*p*=0.755).

The lack of any significant differences in the outcomes observed between the two groups permits the data to be considered as being drawn from a common pool. Thus, from objective refraction, the change in RA was ≥0.50 DC in 52 cases between 1 and 2 months postoperatively and between 2 and 3 months postoperatively, and the corresponding number was 24. For the results arising from subjective refraction, the change in RA was ≥0.50 DC in 36 cases between 1 and 2 months postoperatively and between 2 and 3 months postoperatively, and the corresponding number was 19.

### 3.2. Comparison between 1 and 2 Months Postoperatively

In the 52 cases where objective refraction revealed the change in RA was ≥0.50 DC, the mean (±SD) change in astigmatic power and axis was 0.98 DC (±0.67, 95% CI 0.79–1.17) and 98.6° (±56.5, 95% CI 82.9–114.3), respectively. In these cases, the corresponding changes in astigmatic power and axis at the corneal front and back surfaces over the central 3 mm zone were 1.31 DC (±1.08, 95% CI 1.01–1.60) and 95.1° (±62.3, 95% CI 73.3–111.9) and 2.96 DC (±2.41, 95% CI 2.29–3.63) and 80.0° (±57.2, 95% CI 64.1–95.9). Over the central 5 mm zone, the changes were 1.83 DC (±1.50, 95% CI 1.04–1.74) and 98.4° (±52.5, 95% CI 81.7–117.0) at the front surface and 4.44 DC (±3.32, 95% CI 3.52–5.36) and 91.2° (±48.9, 95% CI77.6–104.8) at the back surface.

The change in astigmatism at the corneal front surface was significantly lower compared with the corresponding change at the back surface (over the 3 mm zone, *p* < 0.001 for power and 0.202 for axis, and over the 5 mm zone, *p* < 0.001 for power and 0.467 for axis).

Comparing changes in corneal surface astigmatism between the central 3 and 5 mm zones, the differences were significant for the front (*p*=0.044) and back (*p*=0.014) surface powers but not for the axes (*p*=0.766 for the front surface and *p*=0.295 for the back surface).

In the 36 cases where subjective refraction revealed the change in RA was ≥0.50 DC, the mean (±SD) change in astigmatic power and axis was 1.03 DC (±0.59, 95% CI 0.83–1.23) and 81.3° (±58.1, 95% CI 61.6–101.0), respectively. In these 36 cases, the corresponding changes in astigmatic power and axis at the corneal front and back surfaces over the central 3 mm zone were 0.69 DC (±0.59, 95% CI 0.45–0.92) and 80.6° (±59.9, 95% CI 56.7–104.6) and 3.48 DC (±3.11, 95% CI 2.43–4.53) and 87.8° (±57.8, 95% CI 68.2–107.4). Over the central 5 mm zone, the changes were 0.93 DC (±0.81, 95% CI 0.67–1.19) and 97.3° (±61.5, 95% CI 77.2–117.4) at the front surface and 4.39 DC (±3.62, 95% CI 3.17–5.62) and 87.7° (±50.5, 95% CI 70.6–104.8) at the back surface.

The change in astigmatism at the corneal front surface was significantly lower compared with the corresponding change at the back surface (over the 3 mm zone, *p* < 0.001 for power and 0.600 for axis, and over the 5 mm zone, *p* < 0.001 for power and 0.461 for axis).

The mean (±SD) pupil size in these cases was 3.36 mm (±0.70, 95% CI 3.13–3.58).

### 3.3. Comparison between 2 and 3 Months Postoperatively

In the 24 cases where objective refraction revealed the change in RA was ≥0.50 DC, the mean (±SD) change in astigmatic power and axis was 1.06 DC (±0.61, 95% CI 0.80–1.31) and 88.1° (±59.6, 95% CI 62.9–113.2), respectively. In these cases, the corresponding changes in astigmatic power and axis at the corneal front and back surfaces over the central 3 mm zone were 0.71 DC (±0.62, 95% CI 0.45–0.97) and 78.5° (±0.43, 95% CI 85.1–127.5) and 2.56 DC (±1.90, 95% CI 1.76–3.36) and 86.2° (±49.3, 95% CI 65.4–107.0). Over the central 5 mm zone, the changes were 1.20 DC (±1.10, 95% CI 0.73–1.66) and 78.5° (±66.7, 95% CI 50.3–106.7) at the front surface and 3.58 DC (±3.58, 95% CI 2.07–5.09) and 90.7° (±59.5, 95% CI 65.6–115.8) at the back surface. The change in astigmatism at the corneal front surface was significantly lower compared with the corresponding change at the back surface (over the 3 mm zone, *p* < 0.001 for power and 0.245 for axis, and over the 5 mm zone, *p*=0.003 for power and 0.499 for axis).

Comparing changes in corneal surface astigmatism between the central 3 and 5 mm zones, the differences were significant for the front (*p*=0.044) and back (*p*=0.014) surface powers but not for the axes (*p*=0.766 for the front surface and *p*=0.295 for the back surface).

In the 19 cases where subjective refraction revealed the change in RA was ≥0.50 DC, the mean (±SD) change in astigmatic power and axis was 0.77 DC (±0.38, 95% CI 0.58–0.96) and 84.0° (±73.3, 95% CI 48.8–119.2), respectively. In these 19 cases, the corresponding changes in astigmatic power and axis at the corneal front and back surfaces over the central 3 mm zone were, respectively, 0.52 DC (±0.44, 95% CI 0.34–0.74) and 96.9° (±55.0, 95% CI 72.1–121.9) and 3.13 DC (±2.72, 95% CI 1.82–4.44) and 109.6° (±56.4, 95% CI 82.4–136.8). Over the central 5 mm zone, the changes were 0.81 DC (±0.57, 95% CI 0.55–1.06) and 88.5° (±56.9, 95% CI 63.0–114.1) at the front surface and 3.24 DC (±2.14, 95% CI 2.21–4.27) and 97.6° (±41.6, 95% CI77.6–117.7) at the back surface.

The change in astigmatism at the corneal front surface was significantly lower compared with the corresponding change at the back surface (over the 3 mm zone, *p* < 0.001 for power and 0.461 for axis, and over the 5 mm zone, *p* < 0.001 for power and 0.511 for axis).

The mean (±SD) pupil size in these cases was 3.67 mm (±1.36, 95% CI 3.08–4.26).

### 3.4. Association between Changes in RA Power (*y*_1_) and Changes in Astigmatic Powers at the Corneal Front (*x*_1_) and Back (*x*_2_) Surfaces

Between 1 and 2 months postoperatively, for the data obtained by objective refraction (*n* = 52), linear regression revealed significant associations between *y*_1_ and *x*_1_ (3 mm zone, *r* = 0.401, *p*=0.003 and 5 mm zone, *r* = 0.326, *p*=0.017) and between *y*_1_ and *x*_2_ (3 mm zone, *r* = 0.325, *p*=0.019 and 5 mm zone, *r* = 0.305, *p*=0.028). The least squares numerical expressions describing the associations between *y*_1_, *x*_1_, and *x*_2_ were

(1) $$\begin{array}{c} 3\text{ mm central zone},y_{1}=0.218x_{1}+0.073x_{2}+0.478, \end{array}$$

(2) $$\begin{array}{c} 5\text{ mm central zone},y_{1}=0.135x_{1}+0.057x_{2}+0.478. \end{array}$$

There were no significant correlations for the data obtained by subjective refraction (*n* = 36, *p* > 0.05).

Between 2 and 3 months postoperatively, there were no significant associations between *y*_1_ and *x*_1_. Significant associations between *y*_1_ and *x*_2_ were found only for the data obtained over the 3 mm central zone. The least squares numerical expressions describing the association between *y*_1_ and *x*_2_ were

(3) $$\begin{array}{c} \text{objective refraction},y_{1}=0.118x_{2}+0.681\left(r=0.467,n=24,p=0.021\right), \end{array}$$

(4) $$\begin{array}{c} \text{subjective refraction},y_{1}=0.072x_{2}+0.545\left(r=0.510,n=19,p=0.026\right). \end{array}$$

### 3.5. Association between Change in the RA Axis and Changes in the Axis of Astigmatism at the Corneal Front and Back Surfaces

Preliminary explorative analysis revealed no significant associations between changes in RA axis and changes in axis of astigmatism at the corneal surfaces. After exploring a variety of transformations of the data, two significant associations were revealed only between the sine of the change in RA axis (*y*_1*i*_) and sine of the change in axis at the corneal front (*x*_1*i*_) and back (*x*_2*i*_) surfaces as follows.

Between 1 and 2 months postoperatively, for the data obtained by objective refraction, over the 3 mm central zone,

(5) $$\begin{array}{c} y_{1i}=0.749-0.303x_{2i}\left(r=0.299,n=52,p=0.031\right). \end{array}$$

Between 2 and 3 months postoperatively, for the data obtained by subjective refraction, over the 3 mm central zone,

(6) $$\begin{array}{c} y_{1i}=0.6614-0.4755x_{1i}\left(r=0.474,n=19,p=0.040\right). \end{array}$$

### 3.6. J_0_ and J_45_ Vectors

Between 1 and 2 months postoperatively, for the data obtained by objective refraction (*n* = 52), linear regression revealed significant associations between the J_0_ vectors describing the change in the corneal front surface astigmatism (*x*) and change in astigmatism according to objective refraction (*y*). The least squares regression lines equating the two vectors were as follows:

(7) $$\begin{array}{c} \mathrm{over}\;3\text{ mm central zone},y=-0.301x-0.034\left(r=-0.441,n=52,p=0.001\right), \end{array}$$

(8) $$\begin{array}{c} \mathrm{over}\;5\text{ mm central zone},y=0.197x-0.020\left(r=0.396,n=52,p=0.005\right). \end{array}$$

For the J_45_ vectors, a significant association between the change in corneal front surface astigmatism (*x*) and objective refraction (*y*) was found for the results obtained over the central 3 mm zone, but not for the 5 mm zone. The least squares regression line equating the two vectors over the 3 mm zone was

(9) $$\begin{array}{c} y=-0.200x-0.096\left(r=-0.284,n=52,p=0.046\right). \end{array}$$

There were no significant associations between J_0_ and J_45_ vectors describing the changes in corneal front surface astigmatism and the changes in astigmatism according to subjective refraction.

No significant correlations were revealed between J_0_ and J_45_ vectors describing changes in the corneal back surface astigmatism and changes in astigmatism according to either objective or subjective refraction.

Between 2 and 3 months postoperatively, linear regression revealed a significant association between the J_45_ vectors describing the change in the corneal front surface astigmatism (*x*) and change in astigmatism according to objective refraction (*y*). The least squares regression line equating the two vectors was as follows:

(10) $$\begin{array}{c} y=-0.462x+0.013\left(r=-0.428,n=24,p=0.033\right). \end{array}$$

No other significant correlations were revealed for J_0_ and J_45_ vectors describing changes in astigmatism at the corneal surfaces with changes in astigmatism according to objective or subjective refraction (*p* > 0.05).

## 4. Discussion

There are reports claiming that differences in the optical design of monofocal nontoric IOLs affect the quality of postoperative vision [39–41]. The current study did not reveal any significant differences between the two IOL designs in relation to the magnitude of changes in the ocular residual astigmatism (RA) revealed by vector analysis over the two postoperative periods. The aim of the study was to determine if there were tangible connections between changes in RA and the posterior corneal surface after routine phacoemulsification, not to investigate the quality of vision. Changes in RA were not related to IOL design.

Changes in corneal front surface astigmatism (FSA) are not uncommon following modern cataract surgery [8, 42–45]. Vector analysis (both polar and Cartesian formats) revealed significant links between changes in RA by objective refraction and FSA as noted in equations (1), (2), and (7)–(10). There were no significant links between changes in RA by subjective refraction and FSA. The mean age of our subjects was 69.5 (SD, ±10.4) years; instances of poor tear stability were not unexpected and may have influenced the assessment of corneal topography. The perceptual component of nonoptical astigmatism, coupled with any inadvertent bias associated with subjective refraction, could also account for the lack of association between RA by subjective refraction and change in FSA.

According to some sources, changes in FSA tend to exceed the astigmatic changes at the corneal back surface [3, 46]. Reports on the corneal back surface astigmatism indicate that the average change is less than 1.00 DC after phacoemulsification [3, 5, 37, 47–52]. The changes in BSA observed in this study exceed the published values. However, this must be viewed with caution because, as noted from the outset, the aim was to quantify the change in BSA in those cases where the unexpected change in RA between periods following cataract surgery was ≥0.50 DC. We did not aim to evaluate the change in BSA resulting just from phacoemulsification.

Glancing at Tables 1 and 2 between 1 and 2 months postoperatively, the change in FSA was greater compared with the change in RA by objective refraction but lower compared with the change by subjective refraction. And the change in BSA was always greater than the change resulting from refraction alone. For the data obtained by objective refraction between 1 and 2 months postoperatively, RA was significantly correlated with both FSA and BSA. However, the least squares numerical expressions describing these associations (equations (1) and (2)) have gradient values much lower than one. The combined changes in FSA and BSA have an impact on RA but not on a 1 : 1 basis. A 1.00 DC change in FSA and BSA will have an impact on RA by less than 0.40 DC. Other factors must be influencing RA besides FSA and BSA.

The modification [34] of the polar method advanced by Alpins [35] revealed significant associations between changes in BSA and RA, but these were not supported by the Cartesian method proposed by Thibos et al. [36]. Statistical noise and limits of accuracy could account for the lack of consistency between the two techniques. Nevertheless, Tables 1 and 2 show that, according to Alpins' technique, the change in RA that developed over the two periods was about 30–45% less than the corresponding change in BSA over the central 3 mm zone. For a 3.00 DC change in BSA, equations (3) and (4) predict changes in RA of 1.04 DC and 0.76 DC, respectively. The implication is clear, and the ratio of change in BSA to change in RA is about 3 : 1. The figures must be read with caution as the figures for BSA are wholly dependent on the specific details, the nuances, of the algorithm entrenched in Orbscan II. It has been claimed that Orbscan II tends to overestimate corneal surface astigmatism when compared with Pentacam [53]. Another study claimed that the results from both instruments are similar, but the results are not interchangeable [54]. A literature search failed to uncover any publication presenting clear, indisputable, unbiased evidence validating (i.e., the difference between the measured and actual astigmatic values) Orbscan II proving it to be inferior compared with Pentacam for the measurement of BSA. Hence, it would be prudent to say, changes in BSA according to Orbscan II technology are linked to changes in RA. Figures 1 and 2 show that in some instances, the change in BSA power was remarkable, and corresponding linear regression equations indicate that change in RA can be predicted from the change in BSA. Thus, according to the data generated by Orbscan II, there is a consistent empirical relationship between changes in RA and corresponding change in BSA evaluated over the central 3 mm of the cornea and, to a lesser extent, over the central 5 mm. The reason for the more significant association between RA and BSA over 3 mm may be related to pupil size. The mean pupil size in our subjects was 3.39 (SD, ±0.68) mm, closely matching values typically encountered under light-adapted conditions [55]. All subjects were refracted, both objectively and subjectively, under normal light conditions. This may account for the more significant association between RA and BSA at 3 mm rather than 5 mm. The abscissa values in equations (1)–(4) exceed the gradient values. This adds further weight to the argument that other factors besides FSA and BSA are influencing RA. The abscissa values in equations (3) and (4) are 0.55 and 0.68. These can be interpreted as the averaged contributions to RA from other factors when the change in BSA is nil. However, the abscissa value in equations (1) and (2) is 0.48, and this is the average contribution to RA by other factors when both FSA and BSA are zero. Factors such as tilting or shifts in the position of the IOL are additional factors contributing to the change in RA. Furthermore, the output of Orbscan II analysis assumes a constant refractive index for the cornea of 1.376. The refractive index of the cornea can vary from subject to subject, and this could be another factor contributing to the abscissa value [56]. Unexpected astigmatic surprises can arise from other factors besides lens rotation after toric IOL implantation [57]. The change in RA may not be fully accounted for by the changes in FSA, BSA, and toric IOL rotation. In such cases, it can be estimated that the average effect on RA resulting from IOL mislocation is about 0.50 DC [57]. This is on par with the abscissa values in equations (1) and (2). The incidence of RA >1.00 DC, after implanting toric IOL without considering measured BSA in the calculation of IOL power, is about 20% [11, 12]. Gao et al. [12] employed a fixed FCA/BCA ratio constant to further refine their estimates for IOL powers, but this did not nullify the incidence of postoperative refractive surprises. Bandeira et al. [11] used Orbscan II to measure corneal front surface astigmatism. If BSA over the postoperative period had been monitored in this study, a better understanding of the impact of any changes in BSA on RA might have been established.

Tables 1 and 2 also show that the mean axis values for the changes in RA, FSA, and BSA were about 90°. This is not surprising considering the range of axes extended from 0° to 180°. However, between 1 and 2 months postoperatively, a significant association between the axis of the change in RA by objective refraction and axis of BSA was found as described by equation (5). And, between 2 and 3 months postoperatively, the subjectively determined change in RA axis was linked to change in FSA axis as described by equation (6).  Figure 3 shows the extent of scatter in the data. Aggregates nested in the top left and lower right of Figure 3 show that, on some occasions, there was almost 90° mismatch between objectively determined change in RA and BSA axes. There may be a statistical link between changes in RA and BSA axes, but it is tenuous. For example, for changes in BSA axes of 0° and 90°, the predicted respective changes in the axes for RA are 49° and 27°. Why should there be a mismatch between the axes? As noted earlier, the consistent abscissa values in equations (1)–(4) point to other factors influencing change in RA power. The same factors are expected to influence the change in axis of RA. The bundles at the top left and lower right of Figure 3 probably arise from the cumulative effects of other factors influencing RA.

## 5. Conclusions

Changes in corneal back surface astigmatism evaluated over the central 3 mm of the cornea were associated with a change in ocular refractive astigmatism in complication-free pseudophakic eyes.

## Figures and Tables

**Table 1:** Calculated change in the astigmatic power (DC, diopters) and axis (°) with reference to the results obtained during objective refraction and Orbscan II analysis of the corneal back surface (CBS) over 3 and 5 mm central zones.

	1 and 2 months (*n* = 52)	2 and 3 months (*n* = 24)

Objective refraction, power (DC)	0.98 (0.67), 0.79–1.17	1.06 (0.61), 0.80–1.31
FSA, 3 mm	1.31 (1.08), 1.01–1.60	0.71 (0.62), 0.45–0.97
BSA, 3 mm	2.96 (2.41), 2.29–3.63	2.56 (1.90), 1.76–3.36
FSA, 5 mm	1.83 (1.50), 1.04–1.74	1.20 (1.10), 0.73–1.66
BSA, 5 mm	4.44 (3.32), 3.52–5.36	3.58 (3.58), 2.07–5.09

Objective refraction, axis (°)	98.6 (56.5), 82.9–114.3	88.1 (59.6), 62.9–113.2
FSA, 3 mm	95.1 (62.3), 73.3–111.9	106.3 (50.3), 85.1–127.5
BSA, 3 mm	80.0 (57.2), 64.1–95.9	86.2 (49.3), 65.4–107.0
FSA, 5 mm	98.4 (52.5), 81.7–117.0	78.5 (66.7), 50.3–106.7
BSA, 5 mm	91.2 (48.9), 77.6–104.8	90.7 (59.5), 65.6–115.8

The mean (±SD) and the lower and upper limits of the 95% confidence interval are shown for those cases where vector analysis revealed the change in astigmatism determined by subjective refraction was ≥0.50 DC over the course of the noted periods. FSA = change in front surface astigmatism, over central 3 and 5 mm optical zones. BSA = change in back surface astigmatism, over central 3 and 5 mm optical zones.

**Table 2:** Calculated change in the astigmatic power (DC, diopters) and axis (°) with reference to the results obtained during routine subjective refraction and Orbscan II analysis of the corneal back surface (CBS) over 3 and 5 mm central zones.

	1 and 2 months (*n* = 36)	2 and 3 months (*n* = 19)

Subjective refraction, power (DC)	1.03 (0.59), 0.83–1.23	0.77 (0.38), 0.58–0.96
FSA, 3 mm	0.69 (0.59), 0.45–0.92	0.52 (0.44), 0.34–0.74
BSA, 3 mm	3.48 (3.11), 2.43–4.53	3.13 (2.72), 1.82–4.44
FSA, 5 mm	0.93 (0.81), 0.67–1.19	0.81 (0.57), 0.55–1.06
BSA, 5 mm	4.39 (3.62), 3.17–5.62	3.24 (2.14), 2.21–4.27

Subjective refraction, axis (°)	81.3 (58.1), 61.6–101.0	84.0 (73.2), 48.8–119.2
FSA, 3 mm	80.6 (59.9), 56.7–104.6	96.9 (55.0), 72.1–121.9
BSA, 3 mm	87.8 (57.8), 68.2–107.4	109.6 (56.4), 82.4–136.8
FSA, 5 mm	97.3 (61.5), 77.2–117.4	88.5 (56.9), 63.0–114.1
BSA, 5 mm	87.7 (50.5), 70.6–104.8	97.6 (41.6), 77.6–117.7

The mean (±SD) and lower and upper limits of the 95% confidence interval are shown for those cases where vector analysis revealed that the change in astigmatism determined by subjective refraction was ≥0.50 DC over the course of the noted periods. FSA = change in front surface astigmatism, over central 3 and 5 mm optical zones. BSA = change in Back surface astigmatism, over central 3 and 5 mm optical zones.

## Data Availability

The data used to support the findings of this study are included within the article.
